# Supplementary material for: End-to-End Protocol for the Detection of SARS-CoV-2 from Built Environments
Source: mSystems. 2020 Oct 6;5(5):e00771-20. doi: 10.1128/mSystems.00771-20 (PMC7542562; doi:10.1128/mSystems.00771-20)
Supplement: TABLE S1 [file mSystems.00771-20-st001.pdf]

Sample	Treatment		ddPCR count(20 Rxn)	uL to ddPCR	Copies/uL	Comments	Average Copies/uL
Zeptometrix-Direct Extract	75C 5 Min -No ProK, No Freeze, No RNase inhibitor-N2 (Day 1)	BioRad One Step Super Mix with IDT Special Primer Probes with Iowa Black	6.8	2	3.4	Poor ddPCR intensity-Incomplete Lysis?	NA
			10	2	5.0		
			14.4	2	7.2		
			15.8	2	7.9		
	75C 5 Min -No ProK, No Freeze, No RNase inhibitor-N2 (Day 2)		20	2	10.0		
			28	2	14.0		
			10.8	2	5.4		
	1:1 Dilution water, 1ul RNase Inhibitor, 3 uL Pro K, 50C 10 min, -80C , 95C 4 min, 4C-N2		90	2	45	Lysis method appears to perform	47.0
			102	2	51		
			90	2	45		
Accuplex SeroCare-Direct	Direct Heat 75C 5 min-N2	BioRad One Step Super Mix with IDT Special Primer Probes with Iowa Black	12.8	2.5	5.1	Partial interference by sample resuspension reagent	5.89
	1:1 dilute with Water Direct Heat 75C 5 min-N2		14.8	2	7.4		
			8.4	2	4.2		
			14	2	7.0		
	1:1 dilution water, Direct Heat 75C 5 min-N1		11.8	2	5.9		
			11.6	2	5.8		
			17.4	3	5.8		

Footnote: Digital droplet qPCR was performed using the BioRad QX200 instrument, the IDT primer/probe set for N1 and N2 with a modified probe quencher of Iowa Black -ZEN/IBFQ (Cat# 10006770). The BioRad One-Step RT ddPCR advanced supermix (1864021) was used as the master mix for ddPCR. Samples were directly lysed using either a direct lysis of 75C for 5 min for the SeroCare Accuplex or a Proteinase k/ freeze thaw -80- 95C 4min for the Zeptometrix standard. Treated samples were analyzed directly by ddPCR in triplicate at 1, 2, 2.5, 3, 5, and 7ul direct input to the reaction. Here we present representative results for 2, 2.5, and 3 μL.
