# Supplementary figures and images for: End-to-End Protocol for the Detection of SARS-CoV-2 from Built Environments
Source: mSystems. 2020 Oct 6;5(5):e00771-20. doi: 10.1128/mSystems.00771-20 (PMC7542562; doi:10.1128/mSystems.00771-20)

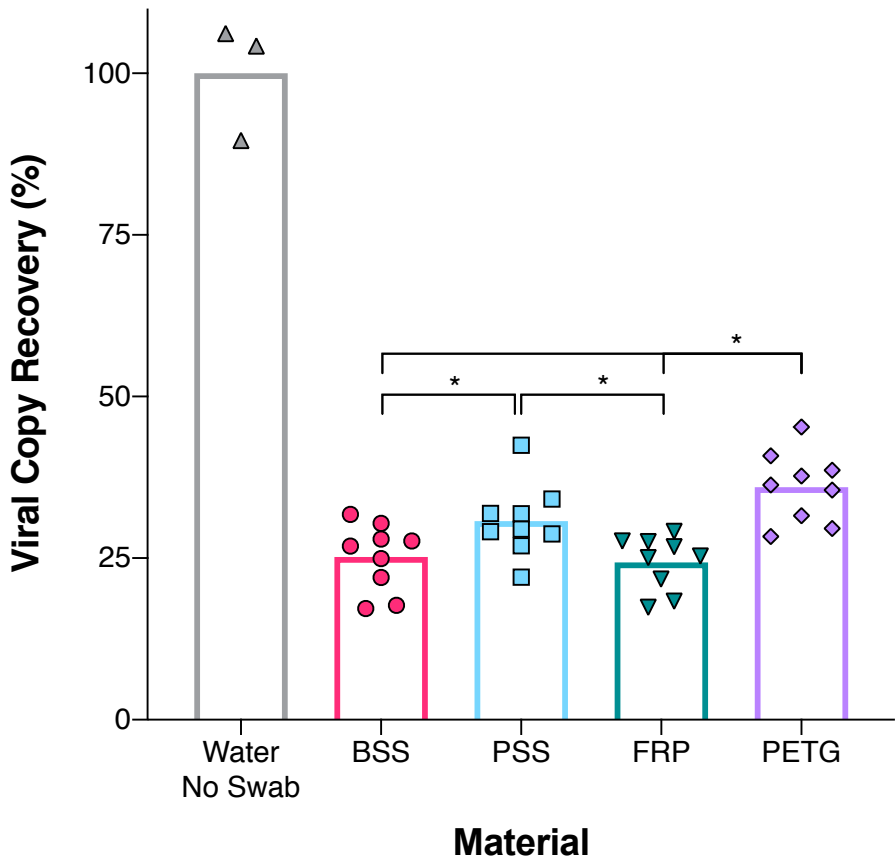

Supplement: FIG S1 [file mSystems.00771-20-sf001.pdf]

A)

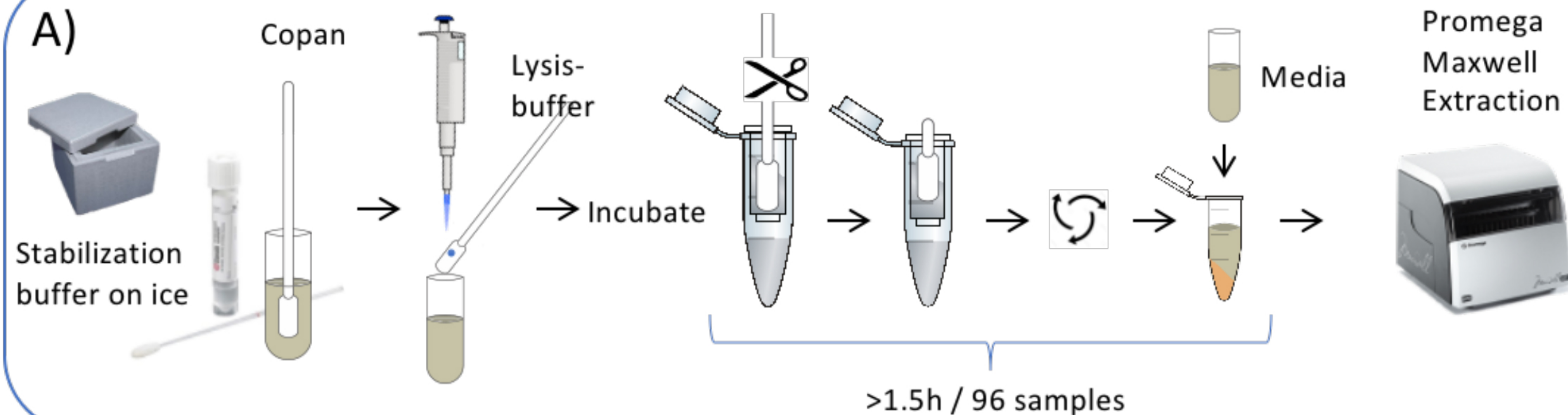

B)

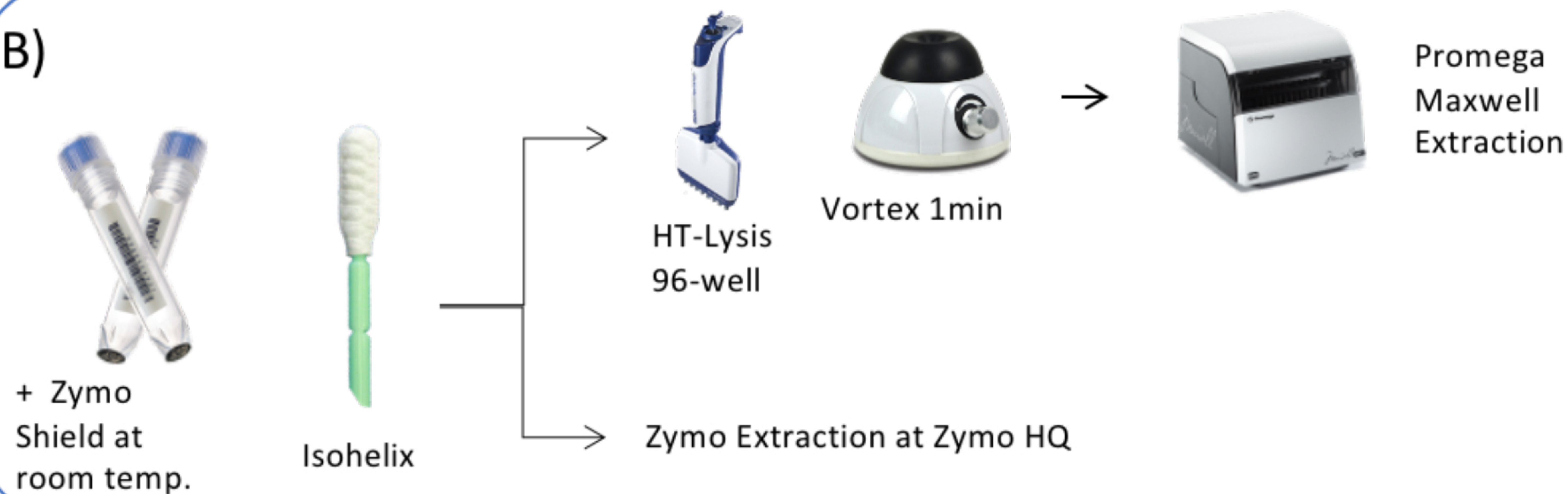

Supplement: FIG S3 [file mSystems.00771-20-sf003.pdf]

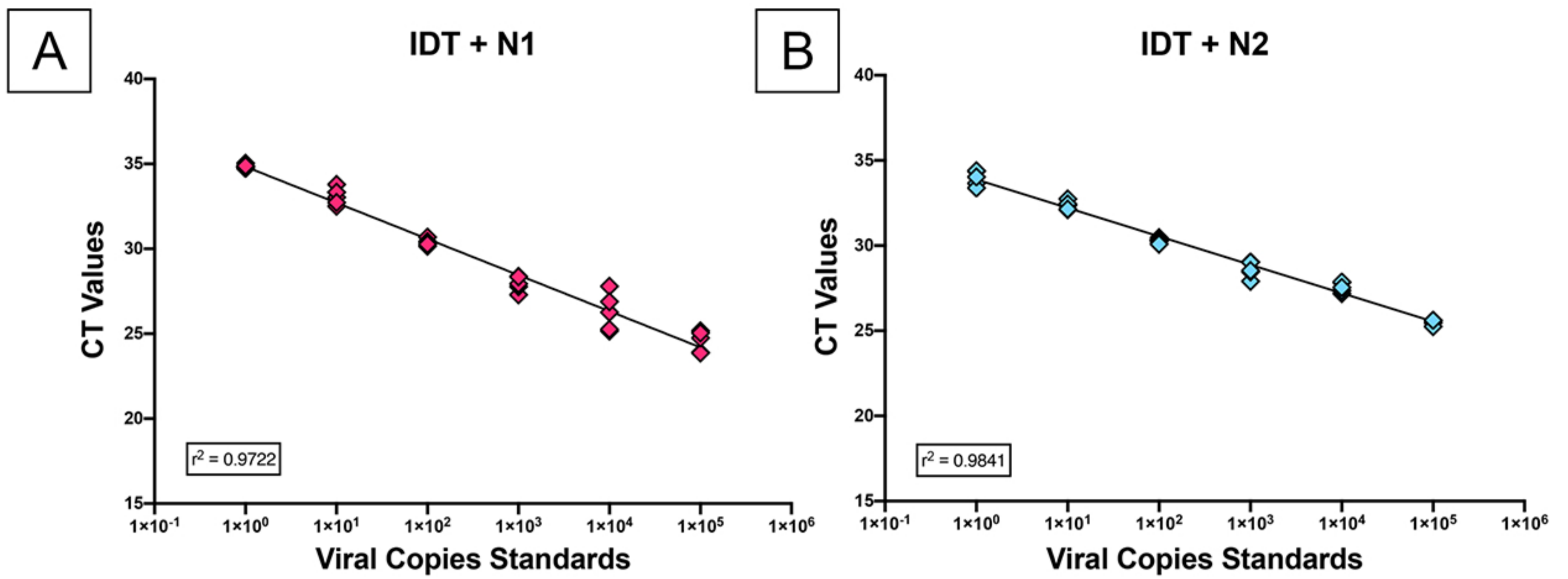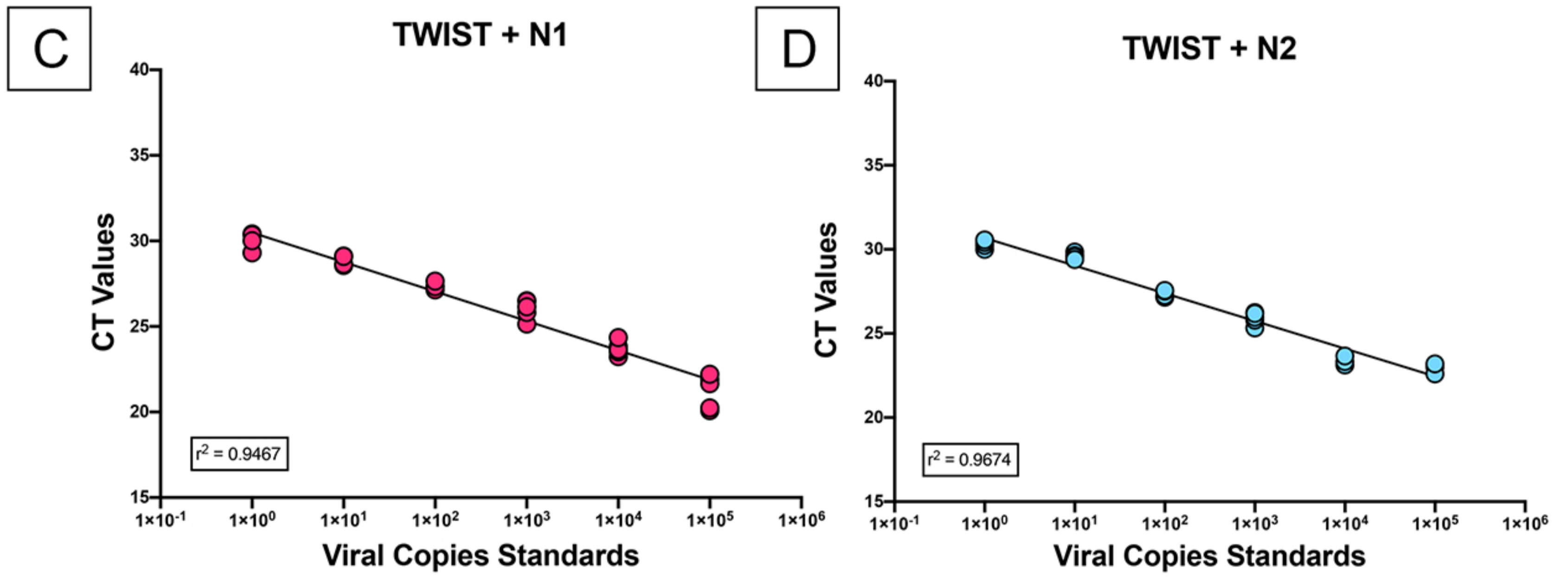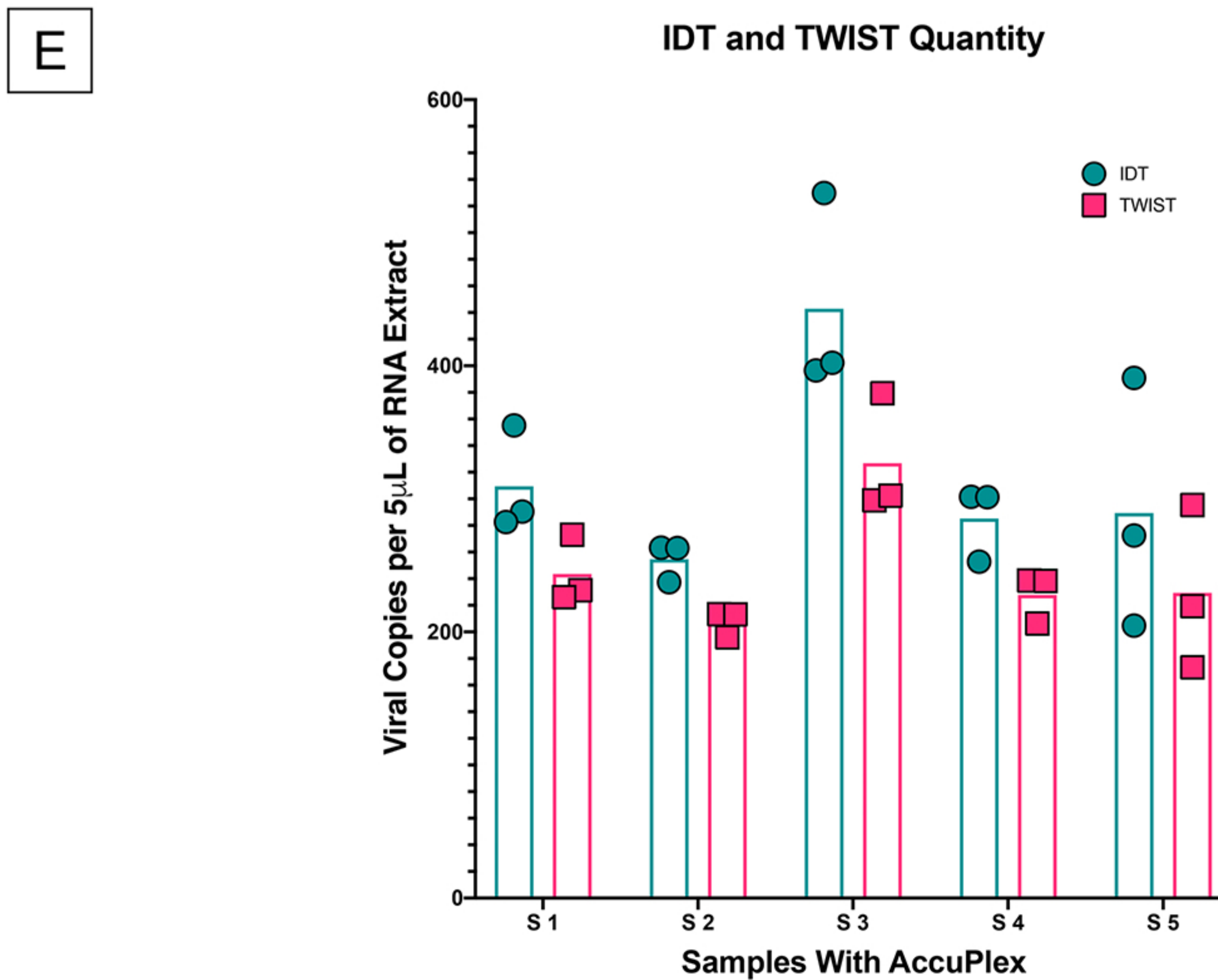

Supplement: FIG S4 [file mSystems.00771-20-sf004.pdf]

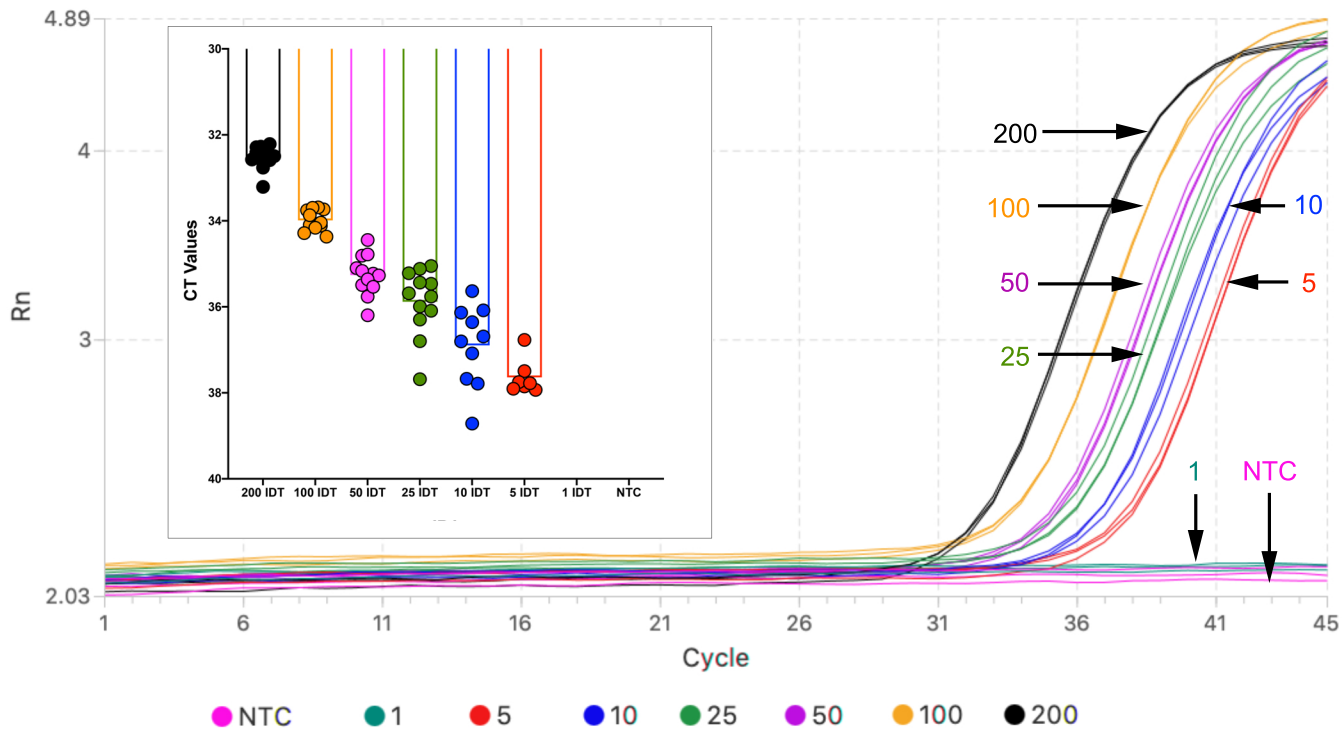

Supplement: FIG S5 [file mSystems.00771-20-sf005.pdf]
