# Supplementary material for: End-to-End Protocol for the Detection of SARS-CoV-2 from Built Environments
Source: mSystems. 2020 Oct 6;5(5):e00771-20. doi: 10.1128/mSystems.00771-20 (PMC7542562; doi:10.1128/mSystems.00771-20)
Supplement: FIG S2 [file mSystems.00771-20-sf002.pdf]

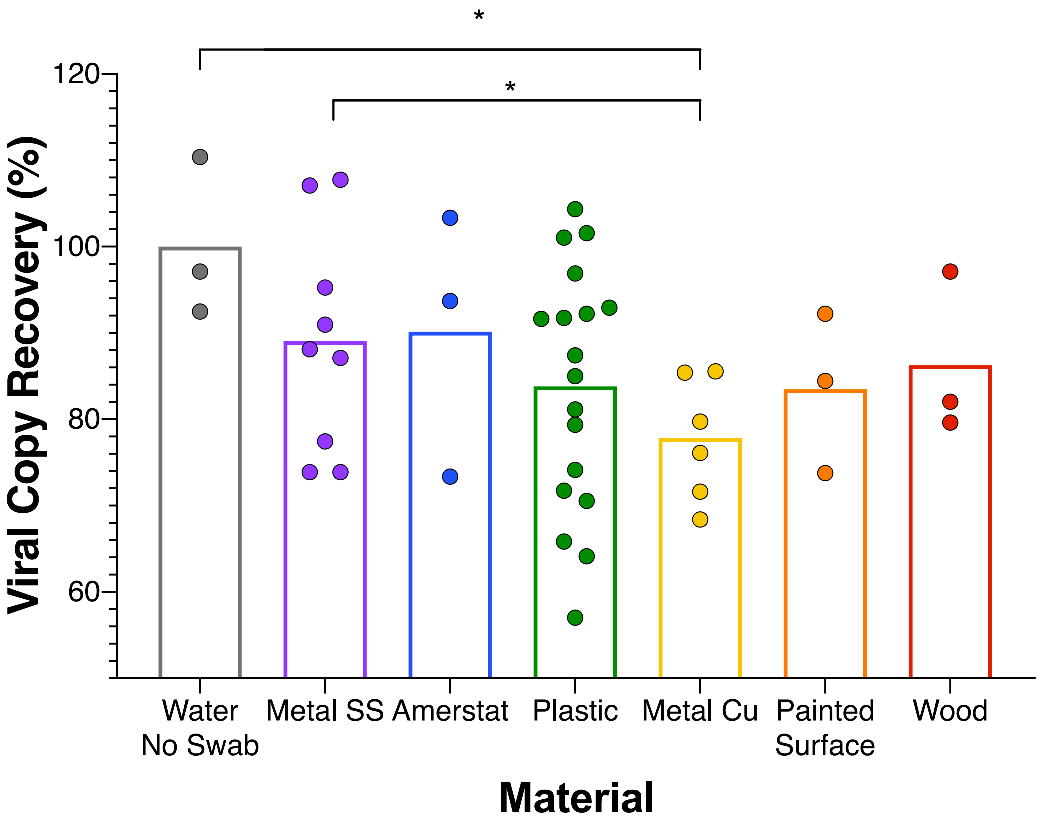
*
*
120
100
80
60
Viral Copy Recovery (%)
Water
No Swab
Metal SS
Amerstat
Plastic
Metal Cu
Painted
Surface
Wood
Material
