# Supplementary material for: End-to-End Protocol for the Detection of SARS-CoV-2 from Built Environments
Source: mSystems. 2020 Oct 6;5(5):e00771-20. doi: 10.1128/mSystems.00771-20 (PMC7542562; doi:10.1128/mSystems.00771-20)
Supplement: DATA SET 1 [file mSystems.00771-20-sd001.pdf]

Data Set-1 document

To validate the first sets of experiments we worked with a second lab to run three additional repeat experiments. The experiments included validation of the first lab's findings for 1) LoD for detection of SARS-CoV-2 using the IDT positive control, 2) extraction efficiency of Accuplex in different media using a Promega Maxwell machine, and manual extraction methods, and 3) synthetic viral recovery from surface materials using a Zeptometrix SARS-CoV-2 positive control applied to four different metals. The second lab followed methods described in the manuscript with a few changes, based on lab setup, as noted below.

Methods for LoD Experiment

Similarly, to experiments run in the first lab, the RT-qPCR LoD assay was performed using the Luna Universal One-Step Reaction Mix, Luna WarmStart RT Enzyme Mix (E3006), Antarctic themolabile UDG, and the N1 primers using the protocol described in the manuscript. The second lab differed by using the Applied Biosystems QuantiStudio 3 qPCR machine instead of the Bio-Rad CFX-96 instrument. Additionally, the second lab increased the number of replicates close to the LoD determined by the first lab, in order to estimate confidence of that LoD. The IDT CDC-EUA SARS-CoV-2 positive control was serially diluted from 200 to 1 copies with the following replicates 200 viral copies (N=5), 100 copies (N=5), 50 copies (N=10), 25 copies (N=20), 10 copies (N=20), 5 copies (N=20), 1 copy (N=8). The CDC program was used for qPCR settings with the addition of a 10 minute UDG step at 25 °C at the beginning of the assay.

Methods for Experiment Testing Extraction Efficiency using Accuplex

For testing extraction efficiency, in the second lab, we employed the same methods described in the manuscript. Briefly, Accuplex SARS-CoV-2 reference material (100 µl, 500 copies) was used for testing the effect that the collection media, collection method, and extraction method have on viral detection. All variables tested were extracted in triplicates. The variables tested included tubes containing 200 µl of DNA/RNA shield (DRS) or nuclease free with and without an Isohelix swab. In addition, two negative controls were used, a negative extraction control using water and a negative control consisting of a swab placed on 200 µl of water and 200 µl of DRS. For samples with swabs, the swab was dipped into the collection media and then 100 µl of Accuplex was pipetted directly into the swab while holding it over the tube, in order to allow the runoff to flow into the tube. Then, the swab was placed in the tube containing the collection media.

Samples were also processed by a manual extraction using Zymo's Quick-DNA/RNA Viral Kit and an automated method using the total Maxwell® RSC Viral Total Nucleic Acid Purification Kit. Prior to addition of the corresponding lysis buffer, all samples were vortexed at full speed for 2 min. For the manual extraction, 400 µl Viral DNA/RNA Buffer was added to each sample, followed by extraction as described by the manufacturer. As for the automated extraction, lysis buffer containing proteinase K was added to each tube, in a 1:1 ratio, and incubated for 10 minutes at room temperature, followed by a 10 minute incubation at 56°C. We transferred all liquid into the appropriate Maxwell cartridge following the Maxwell® RSC Viral Total Nucleic Acid Purification Kit protocol with no deviations. All samples were eluted in 60 µl of nuclease free water.

Immediately after extraction, an RT-qPCR was performed as described above by using 5 µl of the extracted RNA as input for the reaction, with each sample run in triplicates. The viral copy number was quantified by using 10-fold dilutions of the IDT CDC-EUA SARS-CoV-2 positive control (1e5 to 10 copies).

Synthetic Viral Recovery Surface Materials Results

In order to evaluate the viral particle recovery from surface materials, we spotted 10 x 10 µl droplets of Zeptometrix SARS-CoV-2 reference material onto 25 cm^2 of bare stainless steel (BSS), painted Stainless Steel (PSS), polyethylene terephthalate modified with glycol (PETG), and fiberglass reinforced plastic (FRP). Spotted surface materials were placed in sterile petri dishes and dried for 24 hours inside a laminar flow hood. All surface materials were sampled by dipping an Isohelix swab in DRS and swabbing in a raster pattern in different directions, while switching the swab's side midway through swabbing the surface. After swabbing, samples were placed in tubes containing 200 µl of DRS and vortexed at full speed for 2 min. All samples were extracted using the Promega Maxwell and quantified by RT-qPCR as described for the extraction efficiency experiments described above.

Supplemental Results

Overall, the results obtained from experiments in the second lab were similar to the findings in the first lab, including that extraction efficiency was affected by the transport media and whether a swab was present, and that viral recovery varied across materials with the lowest recovery from wood laminate. In the second lab we found that the LoD was 25 copies, when we ran 20 replicates at that dilution, and used a more stringent cutoff of 95% positive identification of those replicates.

LoD Results

In the second lab, we found a good separation of the dilutions, good clustering of replicates (0.59 to 2.6, coefficient of variation), and an acceptable R^2 value of 0.989 (**Supplementary Figure 1**). We were able to recover a positive result for 5/20 samples (25%) for 5 N1 viral copies, 16/20 samples (80%) for 10 N1 viral copies, 19/20 samples (95%) for 25 N1 viral copies and 100 % for all other dilutions. Since 25 copies yielded a positive result on 95% of the replicates, we therefore estimate that this titer is a reliable LOD for this assay.

Extraction Efficiency using Accuplex Results

In order to evaluate how the collection method (swab), collection medium (water, DRS) and extraction method (manual versus automated) influence the detection of viral particles, we independently tested various combinations of these variables, in biological triplicates for extraction, and technical triplicates for qPCR. The Accuplex SARS-CoV-2 reference material was added to tubes containing 200uL of water or DRS, with or without Isohelix swabs. These samples were subsequently extracted followed by RT-qPCR with the N1 primers. The viral copy numbers obtained by the RT-qPCR were compared in order to better understand the effect of each variable on the recovery and detection of viral particles. For this purpose, the average N1 copies obtained from the water suspension, no swab replicates was set as the 100% reference copy number and used to calculate and compare the extraction efficiency among treatments. Overall, we found that the extraction method appears to have the biggest effect on the extraction efficiency, of which a 43.7% significant reduction was observed in the automated extraction ($p < 0.0001$) when using the manual no swab as reference (**Supplementary Figure 2C**). However, independent of the

extraction method, the DRS, with swab had the lowest % viral recovery and detection, 75.9% manual (**Supplementary Figure 2A**) 12.9%, automated (**Supplementary Figure 2B**), but its impact is significantly exacerbated with the automated extractions ($p < 0.0001$) when compared to the water no swab reference (**Supplementary Figure 2B**). DRS did not appear to significantly affect the % viral recovery in either extraction methods when compared to their respective water no swab reference, but a significant difference is observed between DRS, with and without swab ($p <0.0001$) thus indicating that there seems to be a negative additive effect on the viral recovery when using DRS and swab together. Placing the swab in water did significantly reduce the % viral recovery but not as markedly as when the swab was placed on (DRS 83.7% manual, $p = 0.01$, 67.2% automated, $p = 0.002$). Furthermore, it is worth noting that although using DRS showed a reduction in % of viral recovery, samples with DRS exhibited lower coefficient of variation among replicates (ranging from 7.4 to 11.08) than the samples placed in water (ranging from 12.27 to 21.28), independent of the extraction method and usage of swab, with the exception of the automated extraction DRS, swab replicates which showed 54.67 coefficient of variation.

<u>Synthetic Viral Recovery Surface Materials Results</u>

Similar to the Accuplex extraction efficiency experiments, the average viral copy numbers obtained from the water suspension was used as the 100% reference value. As observed previously, the DRS/swab samples yielded a reduced viral recovery (11%) (**Supplementary Figure 3A**). We found that viral recovery varied significantly across different materials (Welch one-way ANOVA, $p<0.0001$), with the highest amount recovered from PSS (2.79%), and PETG (2.4%) followed by BSS (1.4%), while FRP had the lowest recovery (0.42%) (**Supplementary Figure 3B**).

Data Set-1 Figures

Data Set-1 Figure 1. RT-qPCR LoD detection assay indicates the LoD to be 25 viral copies.

Data Set-1 Figure 2. Extraction efficiency for Accuplex in different conditions (including water versus DRS, swab versus no swab, and automated versus manual extraction). Individually DRS, using a swab, and automated extraction all reduced extraction efficiency. These effects were compounded with the most pronounced decrease in yield found when Accuplex was placed in DRS with a swab with automated extraction.

Data Set-1 Figure 3. Viral recovery varied across materials with the highest recovery from PSS followed by PETG, BSS, and finally FRP.

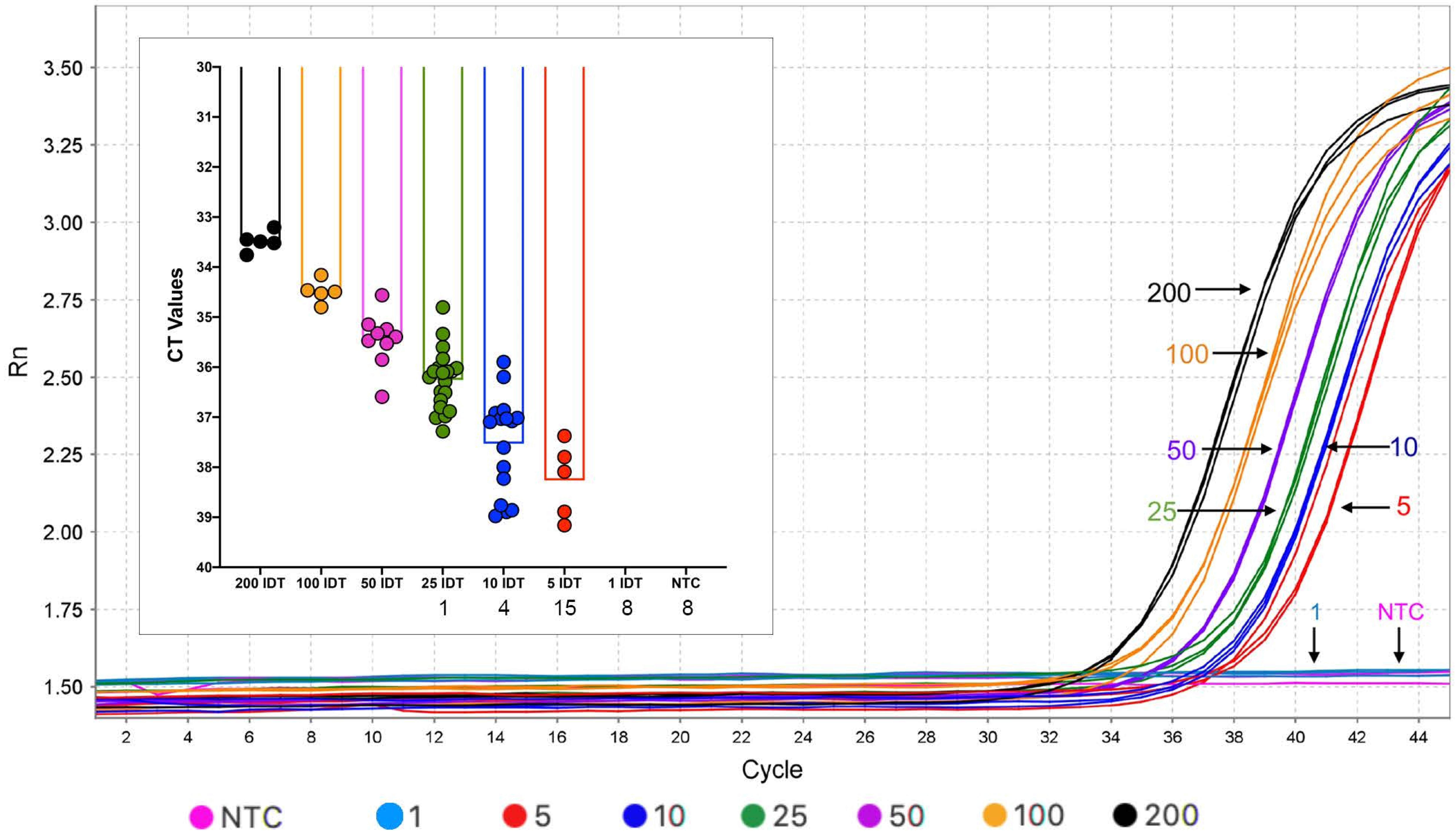

Rn
Cycle
CT Values
200 IDT
100 IDT
50 IDT
25 IDT
10 IDT
5 IDT
1 IDT
NTC
1
4
15
8
8
200
100
50
25
10
5
1
NTC
NTC
1
5
10
25
50
100
200

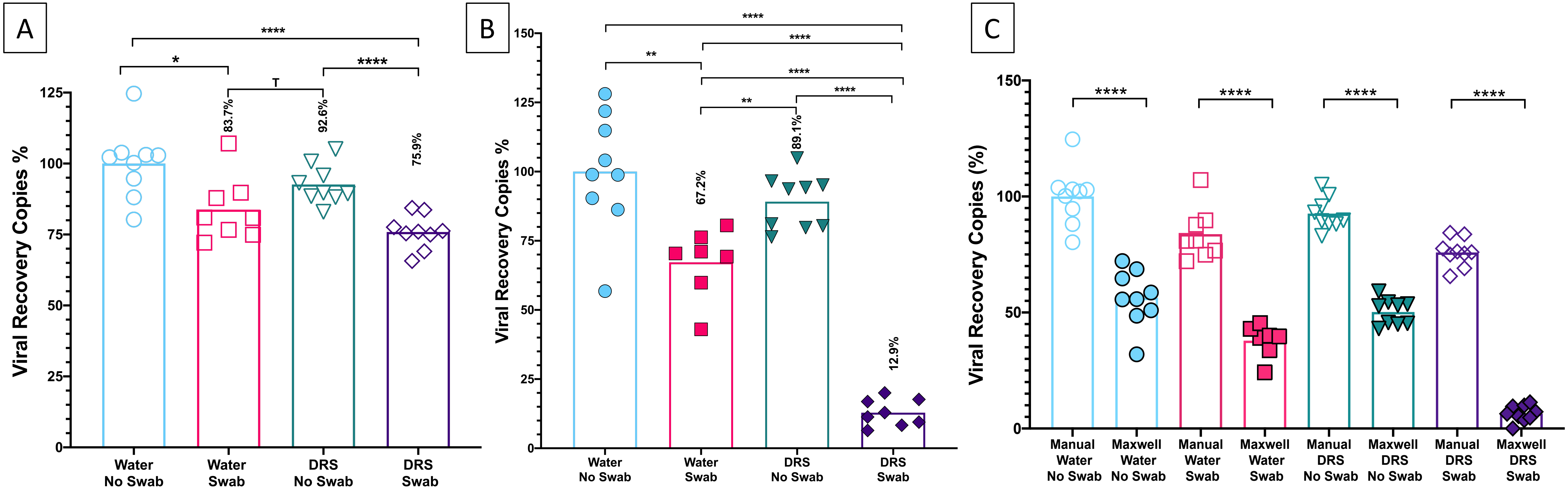

A
B
C
Viral Recovery Copies %
Viral Recovery Copies (%)
83.7%
92.6%
75.9%
67.2%
89.1%
12.9%
*
**
****
T
Water No Swab
Water Swab
DRS No Swab
DRS Swab
Manual Water No Swab
Maxwell Water No Swab
Manual Water Swab
Maxwell Water Swab
Manual DRS No Swab
Maxwell DRS No Swab
Manual DRS Swab
Maxwell DRS Swab

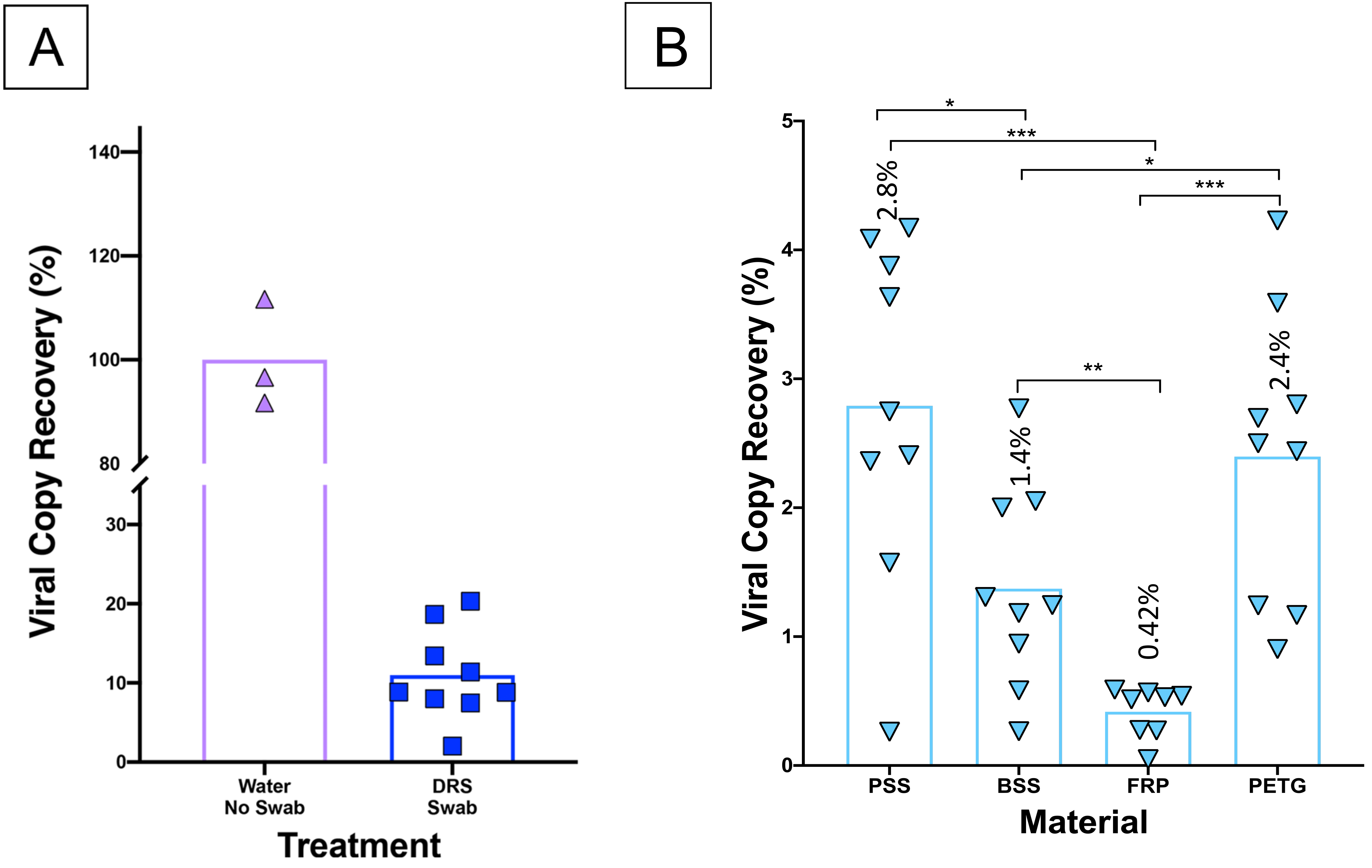
A
Viral Copy Recovery (%)
140
120
100
80
30
20
10
0
Water
No Swab
DRS
Swab
Treatment
B
Viral Copy Recovery (%)
5
4
3
2
1
0
*
***
*
***
**
2.8%
1.4%
0.42%
2.4%
PSS
BSS
FRP
PETG
Material
