# Supplementary material for: End-to-End Protocol for the Detection of SARS-CoV-2 from Built Environments
Source: mSystems. 2020 Oct 6;5(5):e00771-20. doi: 10.1128/mSystems.00771-20 (PMC7542562; doi:10.1128/mSystems.00771-20)

Date	Well	Sample	Target	Conc/µl	Copies Per 20µL Well	Poisson Conf Max	Poisson Conf Min	Sample	Positives	Negatives	Accepted Droplets	Threshold	Copies/µl Sample	RunAve	Standard deviation
7/15/20	C03	NTC	N2	0	0	0.21	0	NTC	0	17125	17125	2090			
7/15/20	E05	APX-Direct-15:15-2uL	N2	0.44	8.8	0.9	0.17	APX-2uL	6	16000	16006	2090	4.4		
7/15/20	E06	APX-Direct-15:15-2uL	N2	0.42	8.4	0.86	0.17	APX-2uL	6	16699	16705	2090	4.2	5.00	1.22
7/15/20	E07	APX-Direct-15:15-2uL	N2	0.64	12.8	1.12	0.32	APX-2uL	10	18434	18444	2090	6.4		
7/15/20	E08	ZEP-Direct-15:15-2uL	N2	4.6	92	5.6	3.7	ZEP-2uL	84	21502	21586	2554	46		
7/15/20	E09	ZEP-Direct-15:15-2uL	N2	5.2	104	6.4	4.2	ZEP-2uL	93	20858	20951	2554	52	48.33	3.21
7/15/20	E10	ZEP-Direct-15:15-2uL	N2	4.7	94	5.7	3.8	ZEP-2uL	88	22168	22256	2554	47		

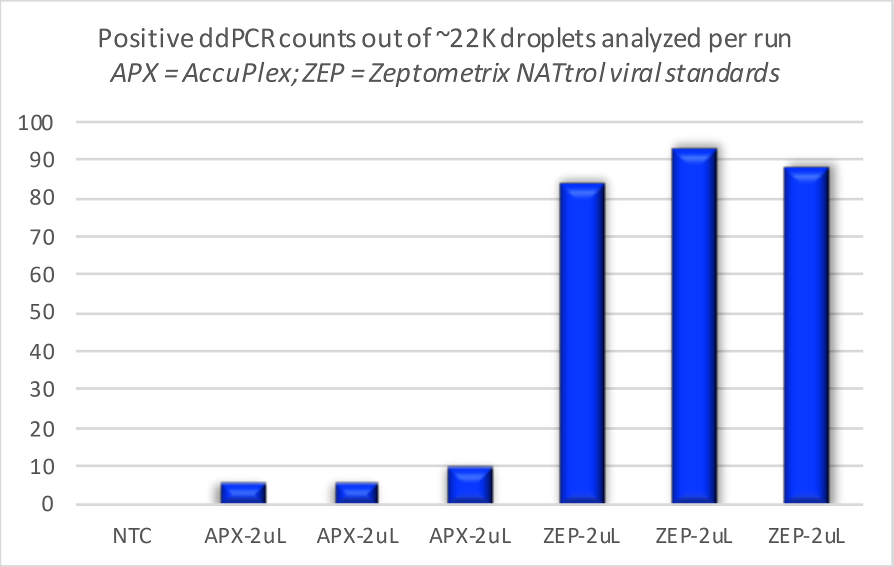

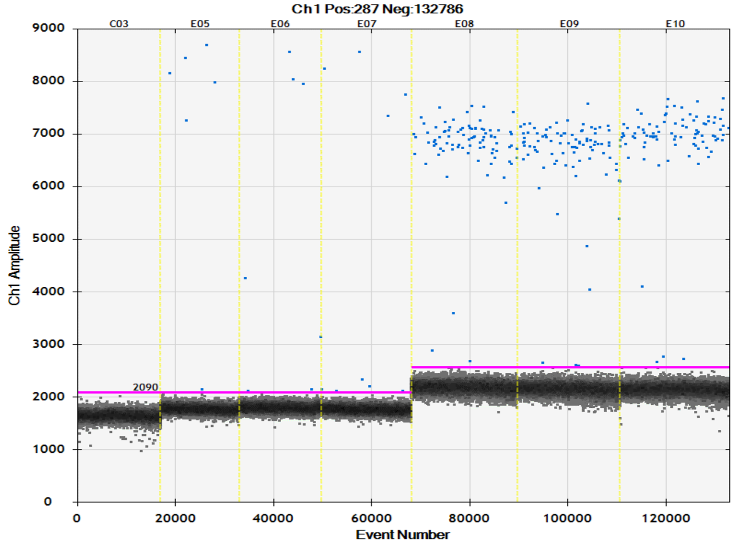

Supplement: TABLE S2 [file mSystems.00771-20-st002.pdf]
