# Supplementary material for: End-to-End Protocol for the Detection of SARS-CoV-2 from Built Environments
Source: mSystems. 2020 Oct 6;5(5):e00771-20. doi: 10.1128/mSystems.00771-20 (PMC7542562; doi:10.1128/mSystems.00771-20)
Supplement: FIG S6 [file mSystems.00771-20-sf006.pdf]

A

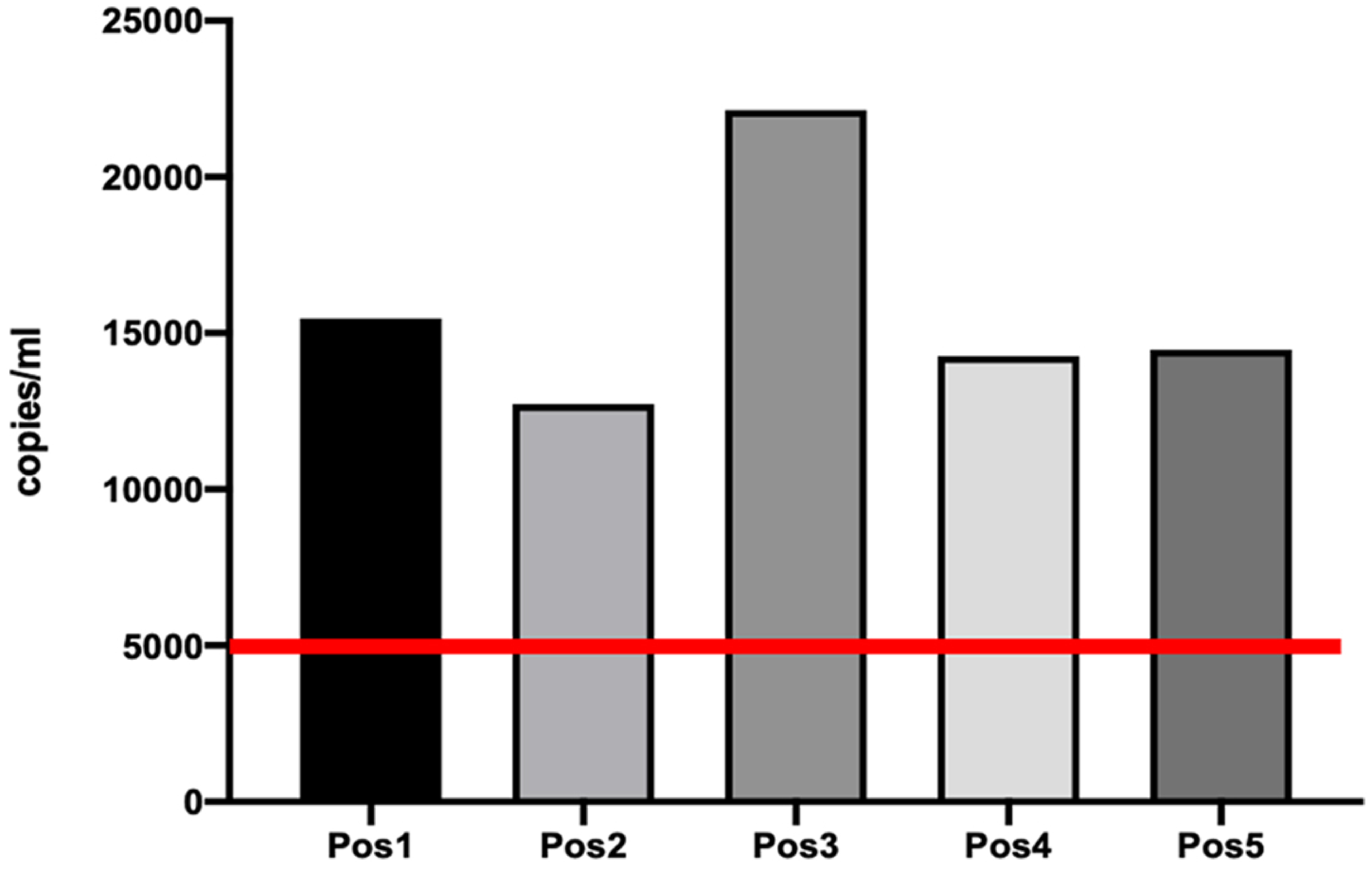

B

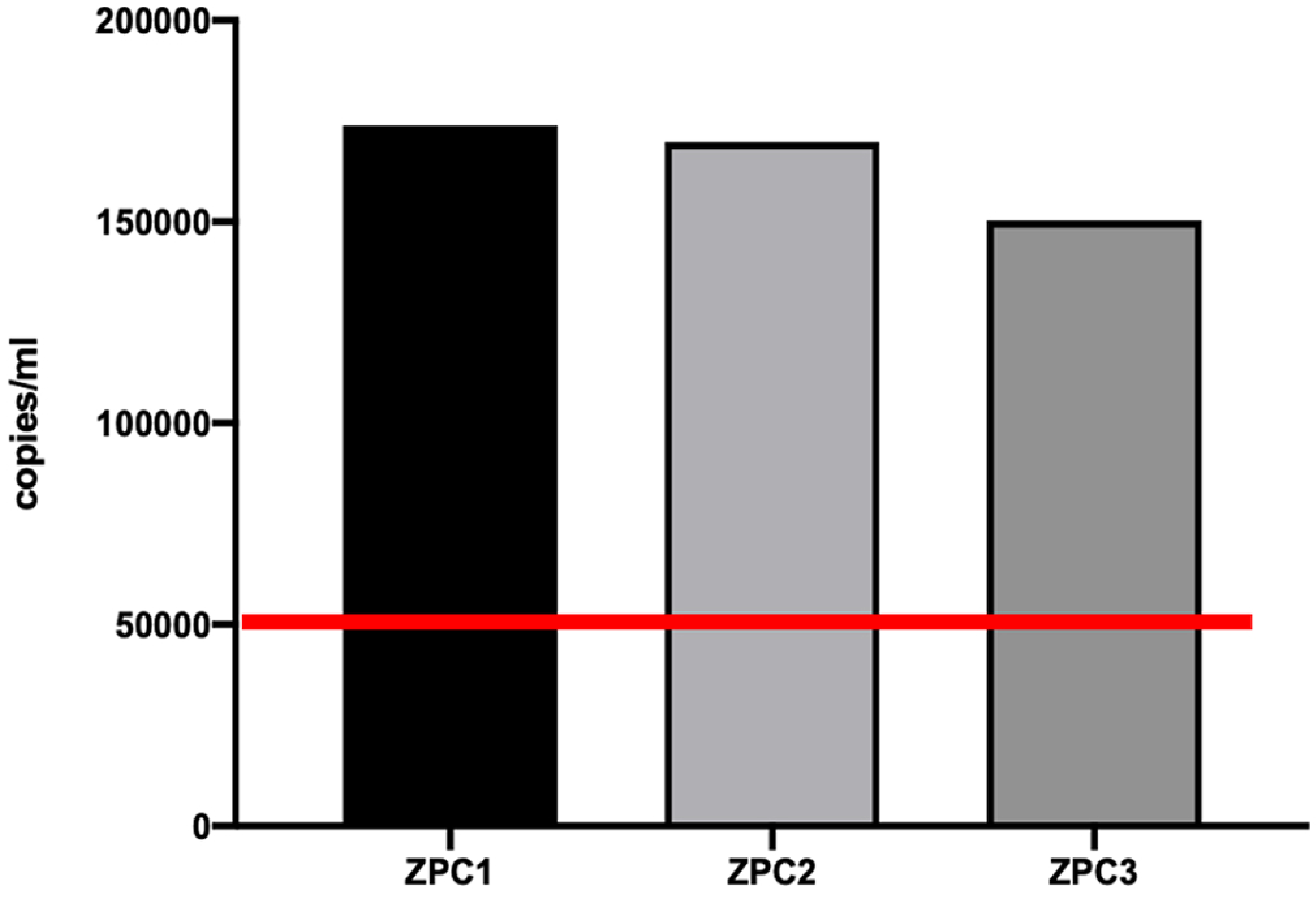

C

N1 Nucleocapsid Fragment Copies per mL

	Manufacturer Reported	ddPCR	RT-qPCR	Fold Difference
AccuPlex	5,000	5,879	15,815	2.69
NATrol	50,000	47,000	164,646	3.5
